# Determination of adequate bony resection margins in inflammatory jaw pathologies using SPECT-CT in primary mandibular reconstruction with virtually planned vascularized bone flaps

**DOI:** 10.1007/s00784-025-06170-2

**Published:** 2025-01-28

**Authors:** Philipp Winnand, Matthias Lammert, Mark Ooms, Marius Heitzer, Marie Sophie Katz, Florian Peters, Stefan Raith, Felix M. Mottaghy, Frank Hölzle, Ali Modabber

**Affiliations:** 1 Department of Oral and Maxillofacial Surgery, University Hospital RWTH Aachen, Pauwelsstraße 30, D-52074 Aachen, Germany; 2 Institute of Pathology, University Hospital RWTH Aachen, Pauwelsstraße 30, D-52074 Aachen, Germany; 3 Department of Nuclear Medicine, University Hospital RWTH Aachen, Pauwelsstraße 30, D-52074 Aachen, Germany

**Keywords:** Inflammatory jaw pathologies, Mandible reconstruction, Medication-related osteonecrosis of the jaw, Osteomyelitis, Osteoradionecrosis, SPECT-CT, Vascularized bone flaps

## Abstract

**Objectives:**

In advanced stages of osteoradionecrosis, medication-related osteonecrosis of the jaw, and osteomyelitis, a resection of sections of the mandible may be unavoidable. The determination of adequate bony resection margins is a fundamental problem because bony resection margins cannot be secured intraoperatively. Single-photon emission computed tomography (SPECT-CT) is more accurate than conventional imaging techniques in detecting inflammatory jaw pathologies. The clinical benefit for virtual planning of mandibular resection and primary reconstruction with vascularized bone flaps has not yet been investigated. This study aimed to evaluate the determination of adequate bony resection margins using SPECT computed tomography (SPECT-CT) for primary microvascular reconstruction of the mandible in inflammatory jaw pathologies.

**Materials and methods:**

The cases of 20 patients with inflammatory jaw pathologies who underwent primary microvascular mandibular reconstruction after the bony resection margins were determined with SPECT-CT were retrospectively analyzed. The bony resection margins determined by SPECT-CT were histologically validated. The sensitivity was calculated as the detection rate and the positive predictive value as the diagnostic precision. Radiological ossification of the vascularized bone flaps with the mandibular stumps was assessed at least 6 months after reconstruction. The clinical course was followed for 12 months.

**Results:**

The determination of adequate bony resection margins with SPECT-CT yielded a sensitivity of 100% and a positive predictive value of 94.7%. Of all the bony resection margins, 97.4% were radiologically sufficiently ossified with the vascularized bone flap and showed no complications in the clinical course.

**Conclusions:**

SPECT-CT could increase the probability of determining adequate bony resection margins.

**Clinical relevance:**

SPECT-CT could have a beneficial clinical impact in the context of primary microvascular bony reconstruction in inflammatory jaw pathologies.

## Introduction

The absence of intraoperative options for rapid bone analysis and for microscopic control of bone margins makes the determination of adequate bony resection margins in mandibular pathologies a current challenge [1–3]. Single-photon emission computed tomography (SPECT) is a nuclear medicine imaging technique in which the distribution of a radiopharmaceutical tracer (e.g., Tc-99m-methylene diphosphonate [MDP]) is recorded with a gamma camera in three different phases that visualize the blood flow, the relative vascular supply, and the bone turnover in pathological bone changes [4]. Using an integrated SPECT computed tomography (SPECT-CT) that fuses SPECT with low-dose CT, morphological and earliest functional bone changes can be visualized [4–6].

The face and its integrity significantly determine a person’s individual appearance [7]. Trauma, tumors, or inflammation can lead to a considerable loss of sections of the mandible [8]. Reconstruction with vascularized bone flaps has been established as the gold standard for the aesthetic and functional rehabilitation of complex facial [9] and mandibular defects [8, 10]. Primary mandibular reconstruction is considered the supreme discipline of oral and maxillofacial surgery, whose fascination is based on the choreographic coordination of two surgical procedures into one [11]. First, the underlying pathology must be completely resected, and vital bony resection margins must be achieved [2, 12], followed immediately by precise reconstruction of the mandibular geometry with vascularized bone flaps [11]. While advances in computer-assisted surgery have steadily improved the efficiency and geometrical accuracy of the reconstructive result [8], the absence of possibilities in routine histology for the microscopic and intraoperative assessment of the bony resection margins remains an unresolved problem in the management of mandibular pathologies [1, 3]. The practical consequences of an intraoperative inability to secure resection margins in bone vary depending on the underlying mandibular pathology.

In reconstruction following *trauma*, the bony resection margins do not require intraoperative assessment, as they are uncritically determined by the bony dimensions of the trauma [13]. The bony resection margins of bone-infiltrating head and neck *tumors* should maintain a safety distance from the tumor margin [14, 15] and are an extremely critical predictor of patient prognosis [16]. However, as these cannot be validated in the operating room without rapid bone analysis procedures [3], primary computer-assisted reconstruction can therefore only be performed at the expense of oncological safety concerns [9]. The bony resection margins in *inflammatory jaw pathologies* do not have to realize oncological safety margins. As orienting feedback, fluorescence-guided surgery can be used to intraoperatively estimate the extent of inflammatory jaw pathologies [4] and the border of necrotic bone [17]. The challenge is to ensure vital bony resection margins without the presence of necrosis or acute inflammation [2, 12, 18]. These can otherwise lead to recurrences of inflammation [1, 19], which can jeopardize the success of the primary bone reconstruction with free flaps [1]. In particular, microvascular reconstruction of the mandible poses a particular challenge in inflammatory jaw pathologies, as chronic inflammatory states and existing comorbidities favor perioperative [12] and postoperative complications [2, 8, 20, 21].

Osteomyelitis (OM), medication-related osteonecrosis of the jaw (MRONJ), and osteoradionecrosis (ORN) are the most common inflammatory jaw pathologies. OM can develop on the basis of odontogenic infections, after untreated or inadequately treated mandibular trauma, after insertion of dental implants and osteosynthesis material (OSM), or after bacteremia [22, 23]. Oncologic systemic therapies with antiresorptive drugs, such as receptor activator of nuclear factor kappa-B ligand (RANKL) inhibitors or bisphosphonates, can lead to osteochemonecrosis [4, 12, 18, 22]. Irradiation of the mandible with > 60 Gy [2, 22] can lead to endarteritis obliterans and subsequent establishment of a cell-, oxygen-, and vessel-depleted environment and full-blown ORN [12, 24]. Despite their different pathways of pathogenesis [22], OM, ORN, and MRONJ hardly differ in their clinical appearance [4, 22] and histological features [23, 25]. 

Detection of inflammatory jaw pathologies using single imaging modalities can be challenging. The orthopantomogram (OPG) provides a fast overview of the mandible but has low sensitivity and low accuracy in the assessment of inflammatory jaw pathologies [5, 6, 26]. Conventional cross-sectional imaging techniques provide an accurate morphologic assessment of the mandible, with the strengths of computed tomography (CT) lying in the detection of sequestration, osteolysis, periosteal reaction and sclerosis, which increases its sensitivity and accuracy compared to OPG [5, 26]. Due to the particularly high sensitivity, the presence of inflammation in the bone can be detected with higher accuracy using nuclear medicine bone scanning methods, such as planar bone scintigraphy [5, 26]. Since planar bone scintigraphy shows osteoblast activity and bone remodeling independently of the underlying pathology [5, 27], planar bone scintigraphy alone has only a low specificity [5, 26]. As the low specificity can be improved by tomographic imaging and the combination of nuclear bone scanning with low-dose CT, SPECT-CT has the highest accuracy in the detection of inflammatory jaw pathologies [5, 6, 26].

In addition to the detection of inflammatory jaw pathologies [4, 5, 18], SPECT-CT can be used for the surgical management of inflammatory jaw pathologies [5, 28]. SPECT-CT has already been used for the postoperative monitoring of vascularized bone flaps in mandibular reconstruction [29–32]. However, virtual planning of mandibular resection and immediate bony reconstruction has been performed mainly with images from conventional imaging modalities such as CT [9, 11]. The evidence-based assessment of mandibular resection margins in inflammatory jaw pathologies is a gap in the literature [22]. The research documented in this paper tested the hypothesis that adequate bony resection margins in inflammatory jaw pathologies can be adequately determined with SPECT-CT. Accordingly, the aim of this study was to evaluate the clinical impact of SPECT-CT for the virtual planning of mandibular resection and primary mandibular reconstruction with vascularized bone flaps in the context of inflammatory jaw pathologies.

## Materials and methods

### Materials

**Fig. 1:**
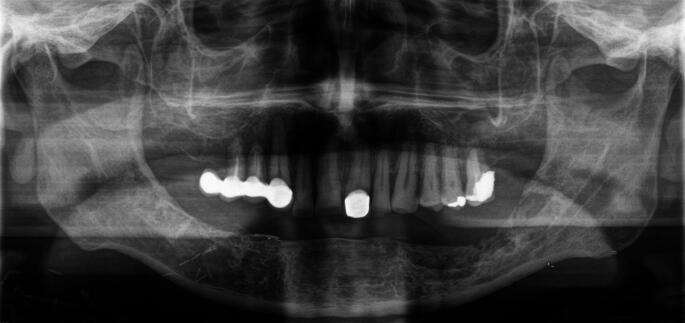
Orthopantomogram (OPG) at the initial presentation of a 53-year-old male patient with extensive osteoradionecrosis (ORN) in the mandible

**Fig. 2:**
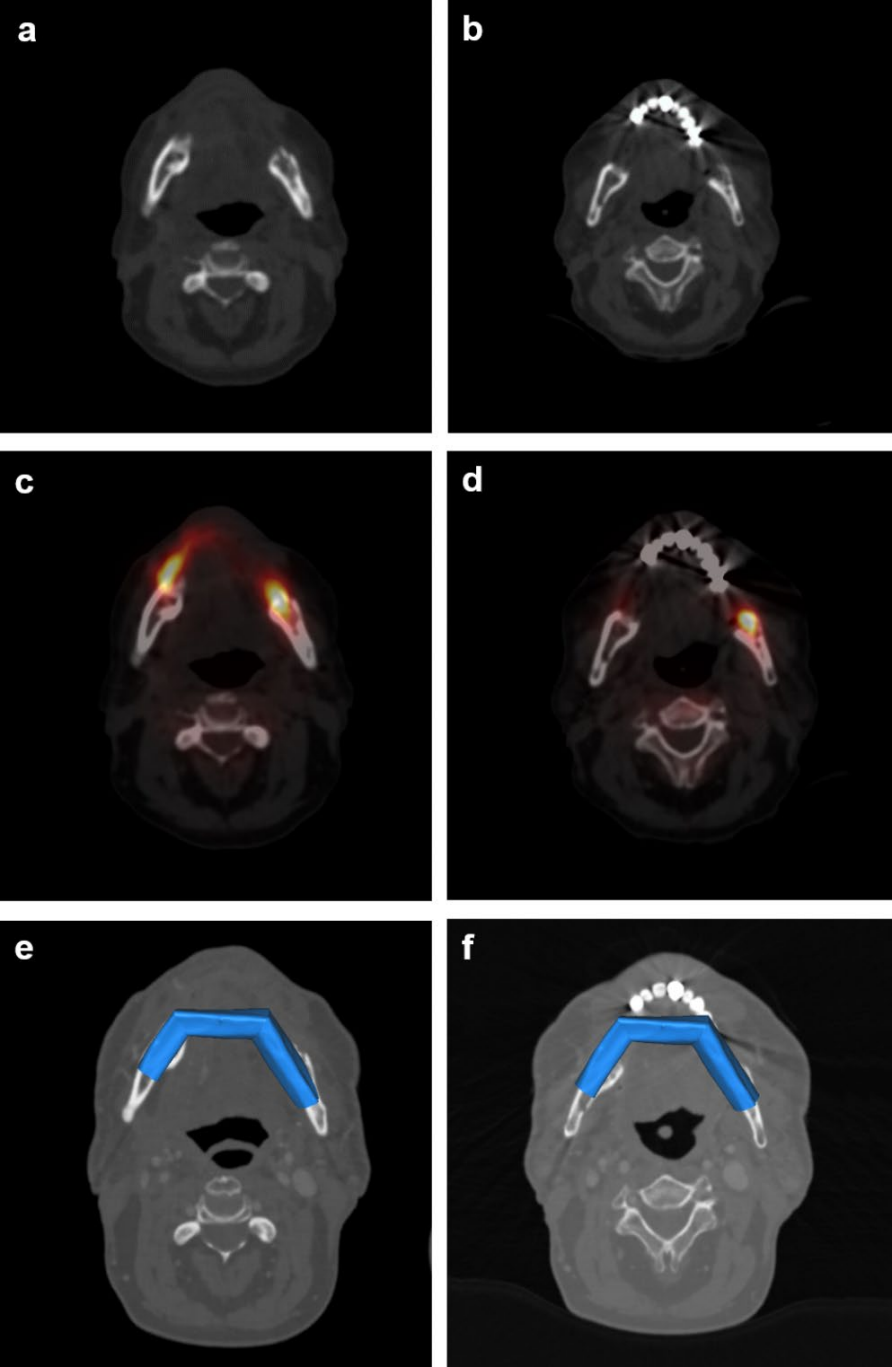
Determination of the right-sided (**a**, **c**, **e**) and left-sided (**b**, **d**, **f**) bony resection margins using low-dose computed tomography (CT) scans (**a**, **b**) and single-photon emission computed tomography (SPECT) scans (**c**, **d**). The osteotomy planes were defined at 2 mm away from abnormal tracer accumulation in SPECT-CT. Virtual planning of primary mandibular reconstruction (**e**, **f**) with a vascularized fibula flap with three segments (blue)

Ethical approval was granted by the Ethics Committee of the Medical Faculty of RWTH Aachen University, Germany (code: EK 24–037; date of approval: 30.01.2024). The cases of 20 patients with inflammatory jaw pathologies (Fig. 1) who underwent primary microvascular bone reconstruction at the Department of Oral and Maxillofacial Surgery of the RWTH Aachen University Hospital between December 2012 and April 2023 were retrospectively analyzed. The bony resection margins were determined by SPECT-CT scans, keeping a distance of 2 mm from abnormal tracer accumulation (Fig. 2). Since the low-dose CT was performed with an image slice thickness of 2 mm, the surgical bone margins for the resection of the inflammatory jaw pathologies were determined with a distance of one healthy (without abnormal tracer accumulation) image slice thickness. The SPECT-CT examinations were performed at the Department of Nuclear Medicine of the University Hospital RWTH Aachen using a Symbia T16 (Siemens AG, Erlangen, Germany) or an Optima NM/CT 640 (GE Healthcare, Chicago, USA). The radiopharmaceutical tracer was 99mTc-hydroxydiphosphonate (HDP). The median tracer activity was 527.5 MBq. All mandibular bone reconstructions were virtually planned and were performed with vascularized bone flaps based on preoperative CT scans. The planning software was ProPlan CMF (Materialise, Leuven, Belgium). The virtual planning and definition of the resection margins were carried out in-house and discussed within the team. STL data was only sent to the company for the fabrication of the cutting guides in all patient cases and additionally for the fabrication of patient-specific implants (PSI) in four patient cases. Segmental fixation of the vascularized bone flaps was performed using conventional OSM (*n* = 16 patients) or PSI (*n* = 4 patients). The OSM was produced by Medartis (Basel, Switzerland).

Inclusion criteria were the performance of disease-related primary bone reconstruction in which the bone was resected due to abnormal tracer accumulation in SPECT-CT, and survival of the vascularized bone flap. Exclusion criteria were an age < 18 years and secondary bone reconstructions in which no abnormal tracer accumulation was detected in SPECT-CT.

### Study design

This study aimed to evaluate the determination of adequate bony resection margins using SPECT-CT in primary microvascular bony reconstruction in the context of inflammatory jaw pathologies. Each contact surface between the vascularized bone flap and the mandibular bone stumps was evaluated separately. In order to compare the resection margins determined by SPECT with the gold standard, the resection margins determined by SPECT-CT were first validated histopathologically. According to routine histology, the bony resection margins were first fixed in 10% formaldehyde. This was followed by 2 weeks of decalcification in 10% ethylenediamine tetraacetic acid (EDTA). The bony resection margins were then embedded in paraffine. The samples were then sectioned and stained with hematoxylin and eosin (H&E). In addition to routine histopathology, H&E sections of the resection margins were evaluated semiquantitatively, according to the histologic criteria for acute and chronic osteomyelitis, by a pathologist. Specifically, a modified histopathological osteomyelitis evaluation score (HOES) [33] with a special focus on osteonecrosis was applied.

Radiological ossification of the vascularized bone flaps with the mandibular bone stumps was evaluated at least 6 months after the primary bone reconstruction, or immediately before osteosynthesis material removal was performed. Radiological evaluation of the bony consolidation of the junction between the mandibular bone stumps and the vascularized bone flaps was performed by assessing the callus formation and the visibility of the interface between the mandibular bone stump and the bone flap according to May et al. [34]. Existing callus formation and reduced visibility of the interface between the mandibular bone stump and the vascularized bone flap in the OPG were evaluated as sufficient bony consolidation. The radiological assessment was performed by one single observer.

The clinical course — in particular, the occurrence of complications, such as fistulas, pseudarthrosis, and pathological fractures at the bony resection margins that were determined by SPECT-CT — was followed until removal of OSM, or for 12 months (in cases where no removal of OSM was performed) after microvascular bony reconstruction. The endpoints of the study were the removal of OSM or a follow-up period of 12 months. All data were extracted from the internal clinical database.

### Statistical analysis

Baseline characteristics were indicated as numbers. The histological, radiological, and clinical evaluations were analyzed separately for each bony resection margin. The sensitivity and positive predictive value of determining adequate bony resection margins using SPECT-CT compared to routine histology (gold standard) were presented as percentages. The sensitivity corresponded to the probability with which an adequate (routine histology) bone resection margin was correctly classified as adequate by SPECT-CT. Accordingly, the sensitivity was calculated as the proportion of bony resection margins correctly classified as adequate by SPECT-CT out of the total of adequate bony resection margins, as validated by routine histology. The positive predictive value indicated the proportion of bony resection margins correctly classified as adequate by SPECT-CT out of the total of bony resection margins classified as adequate by SPECT-CT. The radiological and clinical results of resection margin determination by SPECT-CT were given as the percentages of ossified (radiological) and complication-free (clinical) bony resection margins out of the total number of bony resection margins determined by SPECT-CT.

## Results

### Study data

The patient collective comprised 14 women and 6 men. The median age of the patients was 64 years. There was OM in 6 cases, ORN in 9 cases, and MRONJ in 5 cases. The mandibles were reconstructed with deep circumflex iliac artery (DCIA) flaps in 3 cases and with fibula flaps in 17 cases. Vascularized fibula flaps were raised with a median of 2 segments. The median time between SPECT-CT and primary bony reconstruction was 64 days (Tables 1 and 2).

**Table 1:** Summary

Variable	*Study population (n = 20)*
***Age*** *(years)*	64.0 (18.0)
***Gender*** *(n)*	
Female	14 (70.0%)
Male	6 (30.0%)
***Mandibular pathology*** *(n)*	
ORN	9 (45.0%)
OM	6 (30.0%)
MRONJ	5 (25.0%)
***Type of reconstruction*** *(n)*	
Fibula	17 (85.0%)
DCIA	3 (15.0%)
***Number of segments*** *(n)*	
1	3 (15.0%)
2	11 (55.0%)
3	5 (25.0%)
4	1 (5.0%)
***Segmental fixation of the vascularized bone flaps*** *(n)*	
Conventional OSM	16 (80.0%)
PSI	4 (20.0%)
***Time between SPECT-CT and reconstruction (days)***	64 (44.8)

Legend: DCIA = Deep circumflex iliac artery; MRONJ = Medication-related osteonecrosis of the jaw; OM = Osteomyelitis; ORN = Osteoradionecrosis; OSM = Osteosynthesis material; PSI = Patient-specific implant

Parameters are indicated as numbers (with percentages) for categorical data (gender, mandibular pathology, type of reconstruction, number of segments, and segmental fixation of the vascularized bone flaps) or median values (with interquartile ranges) for metric data (age, and time between SPECT-CT and reconstruction)

**Table 2:** Baseline characteristics of the study population

Number	Sex (F/M)	Age (years)	Diagnosis	Defect localization	Flap Type	Number of segments	Time between SPECT-CT and reconstruction (days)
1	F	36	OM	Mandible right	DCIA	1	32
2	F	54	OM	Mandible left	DCIA	1	49
3	M	62	ORN	Mandible left	Fibula	2	310
4	F	68	MRONJ	Mandible right	Fibula	2	117
5	F	55	ORN	Mandible left	Fibula	2	62
6	F	61	ORN	Mandible right	Fibula	1	102
7	F	75	ORN	Mandible right	Fibula	2	69
8	M	70	ORN	Mandible left	DCIA	2	34
9	F	69	MRONJ	Mandible left and right	Fibula	2	176
10	M	65	OM	Mandible right	Fibula	2	66
11	M	64	ORN	Mandible left and right	Fibula	4	73
12	F	76	MRONJ	Mandible center	Fibula	3	54
13	F	76	MRONJ	Mandible left	Fibula	3	30
14	F	64	OM	Mandible left	Fibula	3	78
15	M	73	ORN	Mandible left	Fibula	2	84
16	F	61	OM	Mandible center	Fibula	3	54
17	F	54	MRONJ	Mandible left	Fibula	2	31
18	F	50	OM	Mandible left	Fibula	2	52
19	M	53	ORN	Mandible left and right	Fibula	3	70
20	F	81	ORN	Mandible left	Fibula	2	28

Legend: DCIA = Deep circumflex iliac artery; F = Female; M = Male; MRONJ = Medication-related osteonecrosis of the jaw; OM = Osteomyelitis; ORN = Osteoradionecrosis

Baseline characteristics are indicated separately for each patient. All patients underwent primary microvascular bone reconstruction in the course of an inflammatory jaw pathology. The bony resection margins were determined by SPECT-CT scans

### Histologic findings

**Fig. 3:**
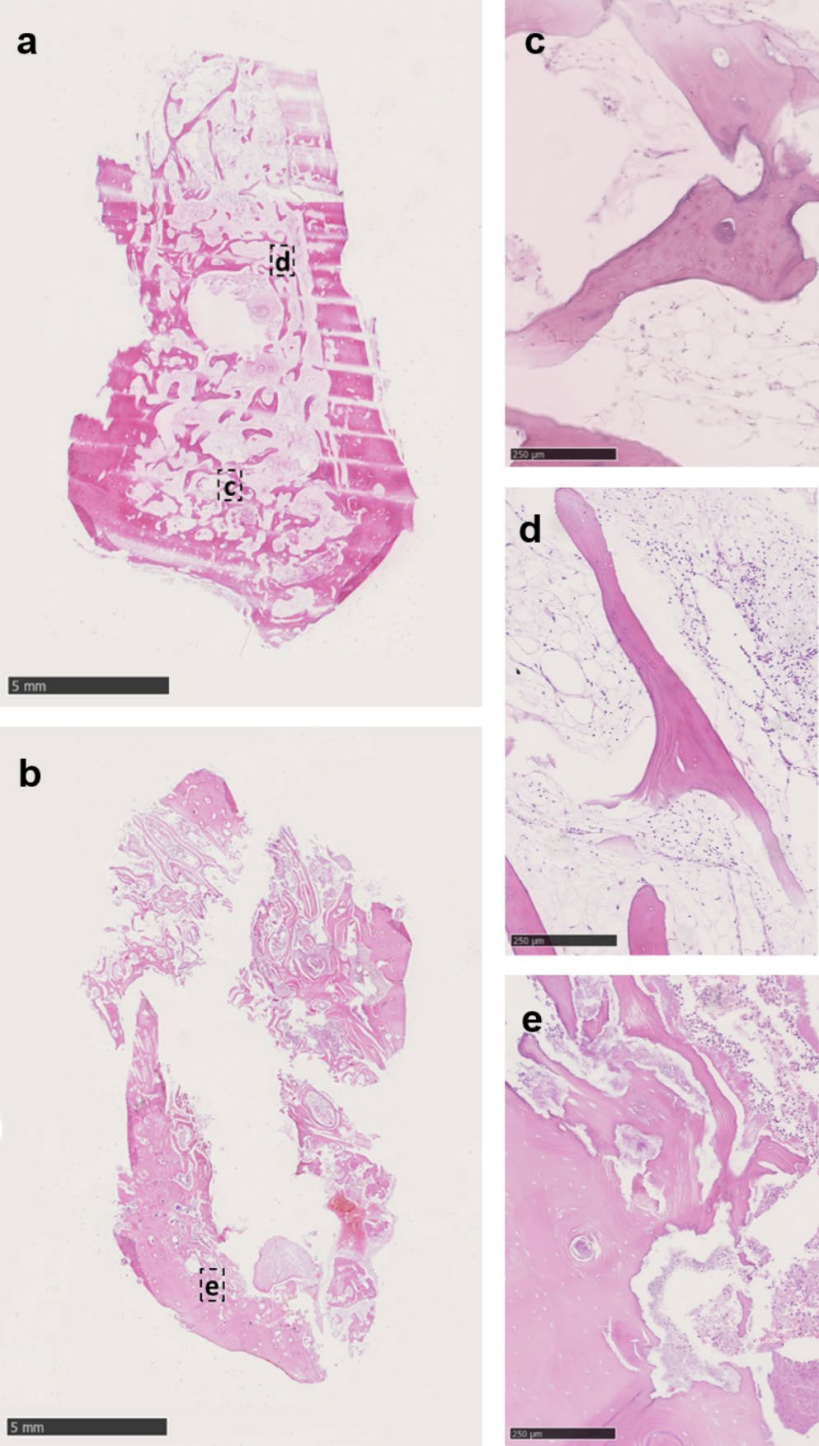
Histological analysis of patient case no. 11 (64-year-old male patient with osteoradionecrosis [ORN] of the right and left mandible). The resection margins determined by SPECT-CT showed no histological signs of necrosis or acute inflammation on the right side. Despite histologic evidence of chronic active OM at the resection margin of the left mandible, ossification was sufficient, and no postoperative complications occurred at the junction between the resection margin of the left mandible and the vascularized bone flap. Two HE-stained transverse sections of the mandibular bone (**a** and **b**) with varying degrees of necrosis, fibrosis, and inflammation, evaluated according to the HOES-grading system; **c**) Selected area of vital bone with smooth contours, preserved nuclear staining of osteocytes, and fatty bone marrow spaces without significant necrosis, inflammation, or fibrosis (HOES: A1-0, A2-0, A3-0, C1-0, C2-0 = no indication of osteomyelitis); **d**) Selected area of bone with smooth contours, but focal absence of vital osteocytes next to marrow spaces with focal replacement of fatty tissue by initially fibrotic, edematous stroma with partial infiltration by lymphocytes, plasma cells, and single neutrophilic granulocytes (HOES: A1-1, A2-0, A3-1, C1-1, C2-2 = signs of subsided, chronic osteomyelitis); **e**) Selected area of necrotic bone with fragmentation, raggedly contoured bone with complete loss of vital osteocytes next to diffusely necrotic marrow spaces with fibrin and dense infiltration by neutrophilic granulocytes (HOES: A1-3, A2-3, A3-3, C1-0, C2-0 = signs of an acute osteomyelitis)

In 2 patient cases, the determination of the bony resection margins using SPECT-CT meant a unilateral resection of the mandible, including the mandibular head. Of the 38 mandibular resection margins available, 2 bony resection margins showed osteonecrosis and soft tissue necrosis (Fig. 3). The bony resection margins determined by SPECT-CT showed no histological signs of necrosis or acute inflammation in 94.7% of all mandibular resection margins (Table 3). The sensitivity of SPECT-CT for the determination of adequate resection margins in bone was 100.0%, and the positive predictive value was 94.7%.

**Table 3:** Histological, radiological, and clinical evaluation of mandible resection margins

Number	Histological evaluation: Bony resection margins without signs of necrosis or acute inflammation?		Radiological evaluation: Ossification ≥ 6 months after primary reconstruction?		Clinical course: Complications within 12 months after primary reconstruction?	
	Right mandible resection margin	Left mandible resection margin	Right mandible resection margin	Left mandible resection margin	Right mandible resection margin	Left mandible resection margin
1	Yes	Yes	Yes	Yes	None	None
2	Yes	**Disarticulated**	Yes	**Disarticulated**	None	**Disarticulated**
3	Yes	Yes	Yes	Yes	None	None
4	Yes	Yes	Yes	Yes	None	None
5	Yes	Yes	Yes	Yes	None	None
6	Yes	Yes	Yes	Yes	None	None
7	Yes	Yes	Yes	Yes	None	None
8	Yes	Yes	**No**	Yes	**Pseudarthrosis**	None
9	Yes	Yes	Yes	Yes	None	None
10	Yes	Yes	Yes	Yes	None	None
11	Yes	**No**	Yes	Yes	None	None
12	Yes	Yes	Yes	Yes	None	None
13	Yes	Yes	Yes	Yes	None	None
14	Yes	Yes	Yes	Yes	None	None
15	Yes	**Disarticulated**	Yes	**Disarticulated**	None	**Disarticulated**
16	Yes	Yes	Yes	Yes	None	None
17	Yes	Yes	Yes	Yes	None	None
18	Yes	**No**	Yes	Yes	None	None
19	Yes	Yes	Yes	Yes	None	None
20	Yes	Yes	Yes	Yes	None	None

Histological, radiological, and clinical evaluations are indicated separately for each mandible resection margin. For histological analysis of the bony resection margins, a modified histopathological osteomyelitis evaluation score (HOES) [33] with a special focus on osteonecrosis was applied. Radiological ossification of the vascularized bone flaps with the mandibular bone stumps was evaluated according to May et al. [34]. Complications included in particular fistulas, pseudarthrosis and pathological fractures

### Radiologic findings at least 6 months after primary reconstruction

**Fig. 4:**
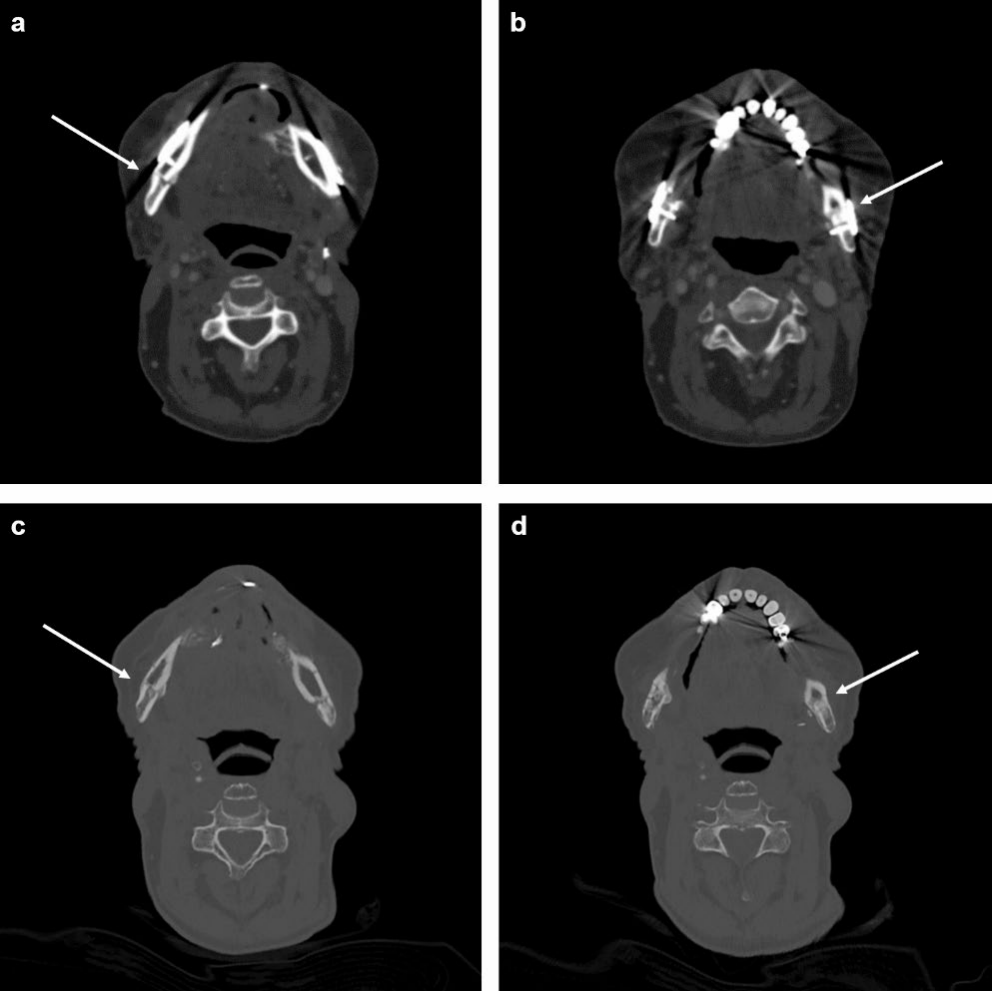
Assessment of the ossification between the vascularized fibula flap segments and the mandibular bone stumps at the right-sided (**a**, **c**) and left-sided (**b**, **d**) resection margins (white arrows) before (**a**, **b**) and after (**c**, **d**) removal of osteosynthesis material

In 97.4% of all bony resection margins, the bony resection margins determined by SPECT-CT were radiologically sufficiently ossified with the vascularized bone flap (Fig. 4). One bony resection margin showed insufficient ossification (Table 2).

### Clinical course up to 12 months after mandibular reconstruction

Of all bony resection margins, 97.4% showed no complications in the clinical course up to 12 months after primary mandibular reconstruction with vascularized bone flaps. The bony resection margin that showed insufficient radiological ossification presented clinically as pseudarthrosis. This specific patient case involved an ORN in the context of radiochemotherapy for a recurrence of a tongue base tumor. This single case differed from the other cases, as wound healing disorders and intraoral exposed bone in the area of the right bony resection margin occurred just a few weeks after the bony reconstruction. Despite multiple modeling osteotomies and wound revisions, there was no clinical improvement, so that pseudarthrosis was clinically suspected. Therefore, intraoperative release of the pseudarthrosis, reosteosynthesis, and interpositionplasty with avascular iliac crest bone were performed. No complications occurred in the further clinical course, and the OSM was removed 11 months later with sufficient ossification.

## Discussion

The adequate treatment of patients with advanced stages of inflammatory jaw pathologies is a fundamental problem in oral and maxillofacial surgery. Based on an algorithmic approach that follows a staged concept, the treatment of early stages of inflammatory jaw pathologies is symptom oriented, noninvasive, and conservative, while advanced stages of inflammatory jaw pathologies may require segmental resection and subsequent reconstruction of the mandible [1, 2, 20]. Although segmental mandibular resection is a prerequisite for the definitive therapy of inflammatory jaw pathologies, radical surgical treatment of inflammatory jaw pathologies is prone to recurrences [1], and subsequent reconstruction of the mandible with vascularized bone flaps is prone to complications [2, 8, 12, 20, 21]. When weighing the surgical treatment options, patient- and disease-specific factors must be considered, as both the patient’s general condition and the surgical site might have been influenced by previous oncological or radiotherapeutic treatment [8, 12, 20]. Although these conditions might particularly affect the success of reconstructive procedures, reconstruction with vascularized bone flaps is an established treatment procedure for OM [11, 22], ORN [2, 8, 20], and MRONJ [2, 8, 12, 21].

While the challenging conditions contrast with the high reconstructive effort involved, the *primary* reconstruction with vascularized bone flaps requires the prior and complete resection of inflammatory bone areas in the same procedure. With the current state of the art, there are unresolved issues related to the intraoperative control and the postoperative analysis of bony resection margins. On the one hand, intraoperative control of bony resection margins is compromised by underestimation of the extent of bony lesions in inflammatory jaw pathologies by both clinical examination [4] and conventional imaging techniques [1]. On the other hand, the value of a histological analysis of bony margins in inflammatory jaw pathologies with regard to surgical treatment planning is highly controversial [22], since — analogous to the similarity of the clinical symptoms [4, 22] — it is also hardly possible to differentiate between OM, ORN, and MRONJ on the basis of histopathological features [23, 25]. The intraoperative analysis and microscopic control of resection margins in bone are impossible without established methods for rapid bone analysis [1, 2]. Therefore, the preoperative use of functional nuclear medicine imaging techniques, such as SPECT, could represent an option that could increase the predictability of adequate bony resection margins in inflammatory jaw pathologies and favor subsequent mandibular reconstruction with vascularized bone flaps.

To understand the potential benefits of SPECT-CT, the limitations of intraoperative visualization options and conventional preoperative imaging methods must be outlined. Fluorescence-guided surgery could provide an intraoperative orientation for the extent and the surgical borders of inflammatory jaw pathologies [4, 17]. However, fluorescence-guided surgery and other intraoperative visualization options cannot be integrated into the workflow of primary mandibular reconstruction with vascularized bone flaps because the virtual reconstruction plan must already be determined preoperatively with the current state of the art. Although conventional preoperative imaging procedures (CT, magnetic resonance imaging, cone beam computed tomography) can be integrated into virtual surgical planning, these are limited to the visualization of morphological changes in the bone without being able to depict functional changes within the bony substance at an early stage [6].

In general, SPECT-CT accurately predicts the extent of inflamed or diseased bone in inflammatory jaw pathologies as compared to clinical examination [4] and pathological findings [18, 35]. Specifically, SPECT determines the (hyper)metabolic activity in OM [5, 23], distinguishes ORN lesions from malignant tumors [24], and identifies different areas within bony MRONJ lesions [18]. Against this background, SPECT can be used for surgical treatment planning in inflammatory jaw pathologies, in particular to determine the extent of mandibular resection and to define the surgical bone margin [5, 18, 28, 35]. There is no study to date that integrates SPECT-CT-guided determination of the bony resection margins into the workflow of computer-assisted mandibular reconstruction. As a significant contribution to the existing literature, this study presents a problem-oriented, in-clinic algorithm that is characterized by the productive synergism of defining the bony resection margins after an accurate imaging of the inflammatory activity in the mandible using SPECT and virtual CT-guided planning of a primary mandible reconstruction with vascularized bone flaps.

The assessment of the bony resection margin status is a matter of debate in all pathologies affecting the mandible [1, 3], as the bone can only be assessed histologically after a decalcification period of at least 8 days [36]. For anticipation, SPECT can be added to surgical planning in oral cavity cancer, as SPECT-assisted detection [37] or exclusion [38] of tumorous infiltration into the mandible allows for improved definition of the (bony) resection margins [39]. While resection margin status correlates with survival rates in oral cancer [16], resection margin control seems to have a decisive influence on the risk of recurrence in inflammatory jaw pathologies. Specifically, non-vitality of the resection margins in ORN and MRONJ was associated with earlier infection recurrence after segmental mandibulectomy, with non-vital resection margins associated with an 11.9-fold increase in infection recurrence compared to vital resection margins [1]. While the definition of the bony resection margins in segmental mandibulectomy without SPECT resulted in non-vital bony resection margins in 19 of 57 patient cases (case-related performance without SPECT: 66.7%) [1], this study demonstrated that the integration of SPECT into bony resection planning (case-related performance with SPECT: 90.0%) could provide a beneficial clinical impact on the determination of adequate bony resection margins in inflammatory jaw pathologies.

Few studies directly associate the bony resection margin status in inflammatory jaw pathologies with the recurrence of inflammation and the clinical outcome [1, 22]. In our study population, we observed only one pseudarthrosis at the resection margins determined by SPECT-CT. The pseudarthrosis occurred in one case where conventional OSM was used for segmental fixation of the vascularized bone flap. The use of PSI to reduce complication rates after mandibular reconstruction with vascularized bone flaps is controversially discussed in the literature [40]. In this study, the influence of OSM used for segmental fixation of the vascularized bone flaps on long-term complications could not be evaluated due to the small group sizes.

The fact that the complication rates after mandibular reconstruction are higher in ORN cases than in non-ORN cases emphasizes the need to take measures to reduce the complication rates of mandibular reconstruction in the course of inflammatory jaw pathologies [41]. With a case-related complication rate of 5% in this study, the determination of the bony resection margins with SPECT seemed to reduce the complication rate compared to microvascular mandibular reconstruction in the context of inflammatory jaw pathologies without SPECT, estimated at 22.5% [21], 33.3% [42], 39.7% [20], and 46% [12]. The fact that our study data showed no association between inadequate resection margins and post-reconstructive complications is consistent with findings in the literature. The bony resection margin status in inflammatory jaw pathologies does not necessarily correspond to symptom control and the clinical course following resection of inflammatory jaw pathologies [22]. A practical explanation for this could be the fact that the blade of the saw has a thickness of 1 mm. From this perspective, the virtually planned distance of the bony resection from the abnormal tracer accumulation in SPECT-CT is added to the thickness of the saw. This could imply that the histologic resection margin status does not necessarily correspond to the status of the resection margin in the situs. Nevertheless, the determination of resection margins using SPECT-CT should aim to ensure that the resection margins determined by SPECT-CT are also validated as adequate in routine histology.

Certain limitations must be considered for this study. These include its retrospective study design with an evaluation of only 20 patient cases, which does not completely rule out low statistical power. Every patient who met the inclusion criteria was included in this retrospective study to minimize selection bias and to achieve a sufficient number of patients to test the hypothesis of this paper. The research design was not standardized with regard to individual patient characteristics, subtype of inflammatory jaw pathology, flap type and number of flap segments. In addition, the time interval between SPECT-CT and primary mandibular reconstruction could not be controlled due to the retrospective nature of the study. The influences of the time interval between SPECT-CT and surgery as well as the inflammatory subtype on the accuracy of the SPECT-CT-guided determination of the bony resection margins could not be sufficiently analyzed in this study due to the small number of patients, but should be the explicit aims of future and prospective studies. Another limitation was the radiological assessment by a single observer. This was an oral and maxillofacial surgeon with experience in the assessment of OPGs.

This study provides an approach to improving the precision of bony resection margin determination through preoperative imaging. The integration of SPECT could impact current surgical workflows as nuclear medicine expertise and specialized diagnostic equipment are required to apply the radiopharmaceutical tracer and perform a SPECT CT. The nuclear medicine specialists could be consulted for virtual planning of the bony resection margins, as nuclear medicine specialists are particularly trained in functional nuclear medicine diagnostics. In the future, the resulting clinical benefit could be increased if imaging could be performed intraoperatively in a manner similar to positron emission tomography (PET) for soft tissue head and neck cancer [43, 44], and if the bony resection margins could be assessed intraoperatively by rapid bone analysis procedures — for example, using spectroscopic optical sensors, as recently demonstrated for bone-infiltrating oral cancer [45, 46]. In this way, the bony resection margins could be modified intraoperatively if required by the surgical situation. Intraoperative modification of the resection margins would undoubtedly pose a challenge for subsequent bony reconstruction, as the virtual plan would have to be changed spontaneously. Since physical cutting guides, which represent the current standard for the implementation of the virtual plan [8, 9, 11], only allow limited flexibility, the use of projected, AR-guided templates could be of great benefit [47, 48].

## Conclusions

SPECT-CT could increase the probability of determining adequate bony resection margins in the context of primary microvascular bony reconstruction in inflammatory jaw pathologies. In this way, SPECT-CT could create the prerequisites for sufficient bony consolidation and a low-complication clinical course, and thus ensure the long-term success of primary bone reconstruction in inflammatory jaw pathologies. As potential clinical implications, the integration of SPECT could help to increase the overall success rate of mandibular reconstructions with vascularized bone flaps and reduce the risk of postoperative complications. Prospective randomized studies comparing microvascular bony reconstructions with and without SPECT-CT are needed in the future.

## Data Availability

The data that support the findings of this study are available from the corresponding author upon reasonable request.
